# The Reform of Out-Patients

**Published:** 1912-08-03

**Authors:** 


### The Hospital

The Workers' Newspaper of Administrative Medicine and Institutional Life, Administration, National Insurance and Health.

No. 1361, Vol. LII. SATURDAY, AUGUST 3, 1912. PRICE ONE PENNY.

### THE REFORM OF OUT-PATIENTS.

The Report presented last week by the Special Committee appointed by the King's Fund to consider the question of the out-patient department generally, with especial reference to the abuse of that department and the suggestions for reform, is a document that deserves the earnest study of all hospital workers in this country. It is not so much because the facts contained in the evidence submitted are new, nor because the recommendations made by the Committee are strikingly original, that the Report requires consideration, but mainly because it is the first time in the history of British hospitals that the subject of out-patients has been dealt with in an authoritative and detailed fashion.

The introduction of the National Insurance Act in hospitals which do not take immediate action will cause a large increase in the activities of the already crowded out-patient departments of our voluntary hospitals. Hence, hospital workers know that this problem is likely to become even more complicated and serious.

It is remarkable that in our newer institutions planned within the last few years the provision for out-patients has been enlarged and amplified beyond the bounds considered desirable a decade ago. Abroad we see the converse tendency. There a perfectly justifiable desire appears to exist to limit the out-patient department, and to create special consultative polyclinics, separate and distinct from the ordinary hospitals, for the treatment of ambulatory cases of the nature dealt with at out-patient departments in this country. With us it is becoming more and more fashionable to send patients indiscriminately chosen from the rank and file to the out-patient department, with the inevitable result that the work of the department is tremendously increased, its efficiency hampered, and the general practitioner, who inevitably suffers from this system of unjustifiable preferential treatment of patients who can afford to pay for medical attendance, irritated and made a potential enemy of an institution to which he should be a friend and auxiliary. The Committee of the King's Fund has fully realised these dangers.

It has drawn attention in the Report just issued to the grave allegations made against the hospital out-patient department by the general practitioners who live in the vicinity of the hospital and who suffer from what one may call, not quite unfairly, the competition instituted by the out-patient departments. We have at various times dealt with this aspect of the problem, and we are glad to find that the Committee agree *in toto* with the strictures we have ourselves passed upon a system that allows no check and no effective control to be exercised over the mass of patients that apply for treatment. Indeed, the suggestions put forward so consistently by The Hospital from the time when the problem of out-patients began to be a problem of urgency are wholly endorsed by the Committee. Efficient organisation of the system under which competent and well-trained almoners are employed to weed out undesirables must be the first plank of any policy of reform. Already the usefulness of the almoner is generally admitted; the pity is that some institutions still regard an untrained inquiry officer, who has had little experience of social conditions, as sufficient. The hospital almoner, to be successful from an institutional point of view, should act in cordial co-operation with the general practitioner. He or she—and it is possible that women here can do much better work than men—should not only investigate the social condition of the would-be patients and their economic standing, but should also consult with the general practitioners in the district, and in that way gain information which would effectually check the wiles of the "black-lister" who now abuses the privileges of out-patient treatment. That being the case, the logical corollary to a system of almoners is the reform of the out-patient department upon what may be called consultative lines. We have always advocated this suggestion, which the Committee of the King's Fund now authoritatively supports after a full consideration of all the evidence. Only by making the out-patient department consultative and by putting the treatment, so far as is professionally possible, in the hands of the private practitioner can the now existing abuses be checked.

Yet a third suggestion, which, again, arises as a necessary corollary from the second, is that the hospital should co-operate with every existing district authority, private or public, for the assistance of the poor. The ideal to be striven for is a co-partnership between hospital special departments, such as the Almoner and the Samaritan Funds, and Poor-Law authority, Charity Organisation, or other philanthropic associations. Such a system of co-operation does not imply that the hospital should lose an iota of its independent work in matters of treatment. It will still retain to the full its power to deal with special cases along special lines, but it will gain the advantage of possessing allies in all directions.

We welcome the Report of the King's Fund Committee in the sincere trust that it will lead hospital authorities to attack the problem of out-patient departmental administration in a radical way. The British Hospitals Association is the proper authority to pave the way for the general dissemination of the suggestions made and for the speedy acceptance of the remedies outlined. The details of the manner in which the suggestions are to be carried out must be discussed by hospital workers in combination, and we hope that the Association will initiate at its next meeting a general discussion. The problem is urgent, and the sooner it is handled the better.

